# Mixtures of *p*,*p*′-DDE, PCB153, BDE47, and PFOS Alter Adipocytic Differentiation of 3T3-L1 Cells

**DOI:** 10.3390/toxics13110975

**Published:** 2025-11-13

**Authors:** Melanie M. Garcia, John L. Pearce, Morgan A. Jacobellis, William S. Baldwin, Kelly J. Hunt, Lisa J. Bain

**Affiliations:** 1 Department of Biological Sciences, Clemson University, Clemson, SC 29634, USA; mmgarci@clemson.edu (M.M.G.); jacobe2@clemson.edu (M.A.J.); baldwin@clemson.edu (W.S.B.); 2 Department of Public Health Sciences, Medical University of South Carolina, Charleston, SC 29425, USA; pearcejo@musc.edu (J.L.P.); huntke@musc.edu (K.J.H.)

**Keywords:** adipogenesis, mixtures, triglyceride, BDE47, PFOS, AMPK, PPARγ, *Fsp27*

## Abstract

Exposure to certain chemicals can promote adipogenesis, but less is known about whether exposure to human-relevant chemical mixtures behaves similarly. Chemical concentrations from the serum of mothers enrolled in the Environmental Influences on Child Health Outcomes—Fetal Growth Study (n = 813) were used in a self-organizing map algorithm to identify five distinct patterns of real-world exposure mixtures containing *p*,*p*′-DDE, PCB153, BDE47, and PFOS. Each mixture profile was exposed to 3T3-L1 cells at 1×, 50×, and 500× serum levels over a 14-day adipogenic differentiation period. Cells were assessed for triglycerides and markers of adipocyte formation and maturation. The results indicate that a mixture dominated by high BDE47 levels (Profile 3) behaved differently than BDE47 alone. BDE47 alone increased expression of *Fsp27*, *Fabp4*, and *Cpt1*, while the Profile 3 mixture inhibited expression of these three genes. In contrast, mixtures dominated by either *p*,*p*′-DDE or PFOS (Profiles 1 and 4) behaved similarly to their dominant chemicals. Exposures inducing Pparγ increased *Fsp27* levels, while those inducing Ampk reduced *Fsp27* levels. These findings suggest that individual chemicals alter transcription factors that control adipogenesis, and the interplay between transcription factors yields synergistic or antagonistic chemical interactions in real-world mixture exposures.

## 1. Introduction

There are over 350,000 anthropogenic chemicals that have been registered for production use [1], of which there are millions of possible chemical combinations that people could be exposed to as a mixture. Investigating the health effects of chemical mixtures is of high importance; however, assessing how chemicals in mixtures interact with one another is challenging [2]. For example, the Center for Disease Control’s National Biomonitoring Program has detected more than 400 potentially toxic chemicals in blood and urine collected from a representative sample of the U.S. population [3]. To assess the health risk of all possible combinations of these chemicals is difficult. Current strategies, such as concentration addition models, are fairly straightforward, but do not capture possible synergistic, antagonist, or non-linear dose–response effects between mixtures of chemicals [4]. Further, these studies often use high concentrations of chemicals and/or only examine chemicals with similar modes of action [5,6,7]. It is of greater interest to develop methods that incorporate human-relevant concentrations of chemical mixtures and focus on examining their effects on a biological-relevant target.

The developing fetus is particularly sensitive to chemicals that mimic hormones, and exposure to these chemicals during critical periods of cell differentiation can lead to adverse effects in adulthood, such as obesity [8]. Obesity has been increasing throughout the world for the past several decades, and studies have suggested that toxicant exposure during fetal development is a factor [8]. Several chemicals can promote adipogenesis and increase the accumulation of triglycerides in adipocytes [9,10,11]. For example, higher maternal BDE serum concentrations were correlated with increased body mass index (BMI) in boys from a longitudinal birth cohort study in California [12]. Additionally, data from seven epidemiological studies reported that maternal serum containing *p*,*p*′-DDE was associated with higher BMI scores in children [13].

Adipocyte formation primarily occurs during fetal development and may represent a window of particularly high susceptibility to chemical exposure. During adipocyte formation, there are two key regulatory transcription factors. The initial factor is C/Ebpα, whose expression increases at the beginning of adipocyte formation [14,15]. C/Ebpα then induces peroxisome proliferator-activated receptor gamma (Pparγ), the master regulator of adipocyte formation [15]. Pparγ is a potential target for chemicals, since its ligand binding site is extremely large, allowing it to accommodate a variety of chemical structures [16]. A binding assay study demonstrated that polar bear PPARγ transfected into 3T3-L1 cells had weak agonist effects with BDE47, and weak antagonist effects with *p*,*p*′-DDE and PCB153 [17]. Additionally, a study that evaluated the ligand binding affinity of PFOS to multiple nuclear receptors, including human, mouse, and rat PPARγ, found that PFOS is a partial agonist of PPARγ [18]. However, one study that evaluated mixtures of chemicals extracted from plastics found that most of their samples did not activate the PPARγ receptor, even though 3T3-L1 cells exhibited increased lipid accumulation [16], indicating that there are other mechanisms for increased adipogenesis. AMPK is another transcription factor that regulates adipogenesis by reducing the function of both C/EBPα and PPARγ [19]. So, a chemical that directly reduces cellular AMPK levels could also result in increases in adipocyte development.

The preadipocyte cell line 3T3-L1 serves as an in vitro model for studying the effects of chemicals on adipogenesis. With an appropriate medium, the cells differentiate from a fibroblast-like phenotype to an adipocyte phenotype. The derived adipocytes have lipid droplets containing triglycerides, and express key adipogenic genes such as peroxisome proliferator-activated receptor gamma (*Pparγ*), fatty acid-binding protein 4 (*Fabp4*), lipoprotein lipase (*Lpl*), carnitine palmitoyl transferase 1 (*Cpt1*), and fat-specific protein 27 (*Fsp27*) [20,21,22]. Exposure to individual chemicals, such as PCB153, has been found to induce abnormal lipid accumulation in 3T3-L1 pre-adipocyte cells [23]. BDE47 exhibits bidirectionality in which the highest and lowest concentrations of BDE47 cause an increase in the expression of *Pparγ* [24]. PFOS induces adipogenesis, in part, by increasing PPAR*γ* activation and lipid accumulation in adipocytes [25]. *p*,*p*′-DDE also increases lipid accumulation and increases adipogenic differentiation markers such as *Fabp4* [26].

Although there are many publications describing the effects of individual chemical exposures on 3T3-L1 cells, fewer studies have examined the effects of chemical mixtures on adipocyte differentiation. One such study determined that 11 plastic mixtures from consumer products, such as water bottles and coffee cup lids, exhibited increased lipid accumulation levels in 3T3-L1 cells [16]. Another study evaluated chemical mixtures found in household dust, showing that 10 of their 11 samples exhibited an increase in lipid accumulation in 3T3-L1 cells [27]. Similarly, a study examining combined mixtures of 26 different chlorinated and brominated chemicals showed increased lipid content in 3T3-L1 cells [28].

The objective of this study is to illustrate a toxicological framework that supports the evaluation of real-world chemical mixtures. To achieve this goal, we build upon a previous epidemiologic mixtures study [29] where exposure mixtures observed in maternal serum at 8–12 weeks gestation were associated with variation in childhood body mass index (BMI) at 4–8 years of age. The exposure data described in Pearce et al. (2021) [29] were used to identify distinct combinations of a simple mixture consisting of four chemicals: 1,1-dichloro-2,2-bis (p-chlorophenyl)-ethylene (*p*,*p*′-DDE), 2,2′,4,4′,5,5′-hexachlorobiphenyl (PCB153), 2,2′4,4′-tetrabromodiphenyl ether (BDE47), and perfluorooctane sulfonate (PFOS). Then, these mixture profiles were used to assess 3T3-L1 adipocyte differentiation, triglyceride accumulation, and transcription factor expression and transactivation in assays designed to examine single chemical and multi-chemical effects.

## 2. Methods

### 2.1. Chemicals

The chemicals used in this study were dichlorodiphenyldichhloroethylene (*p*,*p*′-DDE) (#P-027N, 99.9% pure, Llot # 28464, AccuStandard Inc., New Haven, CT, USA), 2,2′,4,4′-tetrabromodiphenyl ether (BDE47) (#BDE-047S,100.0% purity, lot # 223021176, AccuStandard Inc.), 2,2′,4,4′,5,5′-hexachlorobiphenyl (PCB153) (#C-153S-TP, 99.9% pure, lot # 222051165, AccuStandard Inc.), perflurooctane sulfonate (PFOS) (#77282, 98% pure, lot # BCBX5798, Sigma Aldrich, St. Louis, MO, USA), rosiglitazone (#R2408, 98% pure, lot # 0000108204, Sigma Aldrich), and dimethyl sulfoxide (DMSO) (#BP231-100, 99.7% pure, lot #234276, Fisher, Waltham, MA, USA).

### 2.2. Chemical Mixtures

The exposure mixture ratios used in this study were derived from exposure biomarker data collected as part of the Environmental Influences on Child Health Outcomes—Fetal Growth Study (ECHO–FGS) [29,30]. Specifically, maternal serum concentrations for our four chemicals of interest were analyzed using self-organizing maps (SOMs) to identify five distinct patterns of real-world exposure mixtures (also known as mixture profiles) that occurred in our study population. The resulting mixture profiles were then used as a baseline for our toxicological assessment (Table 1). Individual chemicals were dissolved in DMSO to create a stock solution and stored in amber glass vials at −20 °C. Stocks were diluted with cell culture medium to reach a concentration of 500 times (500×) the calculated serum concentration, with a maximum of 0.5% DMSO added in the cell culture medium. The 500× mixture was serially diluted to create a 50× and 1× mixture (Table 1). For testing individual chemicals, the chemicals were used at the highest 500× concentration. The mixtures at 1× represent real-world serum chemical concentrations in the mothers. The 50× mixture represents the high end of maternal chemical concentrations, while the 500× mixture represents a high level designed to test potential mixture effects and account for extreme chemical serum levels.

### 2.3. 3T3-L1 Cell Differentiation and Chemical Exposure

3T3-L1 pre-adipocyte cells (Zen Bio, Durham, NC, USA) were cultured in DMEM media with 4.5 g/L glucose (Corning, Corning, NY, USA), 10% *v*/*v* bovine calf serum (Sigma-Aldrich), 1% *v*/*v* L-glutamine (Thermo Fisher, Waltham, MA, USA), and 1% *v*/*v* penicillin streptomycin (Thermo Fisher) in a humidified incubator at 37 °C and 5% CO_2_. Cells were passaged at 70–80% confluency and media were changed every 2–3 days. To induce differentiation and adipocyte formation, 3T3-L1 cells were seeded at 6 × 10^4^ cells/well in a Greiner Bio One 24-well plate. On day 0, the medium was replaced with differentiation medium (DMEM with 4.5 g/L glucose, 1% *v*/*v* penicillin streptomycin, 1 µg/mL insulin (Life Technologies, Carlsbad, CA, USA), and 0.5 mM isobutyl-methyl-xanthine (IBMX, Sigma Alrich), and one of the following individual chemicals or mixtures: 0.5% DMSO (control), 2 µM rosiglitazone (positive control) [31,32], 1×, 50×, and 500× concentrations of the chemical Profiles 1–5 (Table 1), or 500× of the highest individual chemical (n = 4–6 replicates per treatment).

On day 2, the differentiation media were removed and replaced with maintenance media (DMEM with 4.5 g/L of glucose, 10% *v*/*v* FBS, 1% *v*/*v* penicillin streptomycin, and 1 µg/mL insulin) containing the same individual chemicals or mixtures. The maintenance media were exchanged every 2 days for a total of 14 days [33] and the test chemicals were replenished with each medium swap.

### 2.4. LDH Cytotoxicity Assay

The CytoTox One (Promega, Fitchburg, WI, USA) fluorescent assay was used to measure LDH release into the medium as an indicator of cytotoxicity. A 100 µL aliquot of medium was collected on day 14 from every exposure well and transferred to a Corning black 96-well plate. CytoTox reagent (100 µL) was used to assess LDH levels in the medium by fluorescence (560 nm excitation, 590 nm emission) on a Synergy H1 hybrid plate reader (BioTek Instruments Inc., Winooski, VT, USA). An additional set of untreated day 14 cells were lysed and LDH levels analyzed (n = 4) to determine fluorescent values for 100% LDH release into the medium. The replicates for each chemical exposure (n = 4) were averaged to calculate the average percent LDH release as a measure of cytotoxicity.

### 2.5. AdipoRed Assays for Triglyceride Accumulation

Lipid accumulation in the same day 14 cells was measured using AdipoRed (Lonza, Basel, Switzerland), which stains triglycerides, and NucBlue (Thermo Fisher), which stains the DNA in nuclei and is used as a proxy for the total number of cells in each well. To start the assay, cells were rinsed with PBS and then a mixture of 4% AdipoRed and 2.4% NucBlue in 1× PBS was incubated with the cells for 25 min. Accumulation of NucBlue (360 nm excitation, 460 nm emission) and AdipoRed (485 nm excitation, 572 nm emission) was measured by fluorimetry on a Synergy H1 plate reader (BioTek). Readings were taken using the area scanning function to measure 21 points across one well, which were averaged to determine NucBlue and AdipoRed fluorescence values per well (n = 4 wells per exposure group). Images of the cells were captured on a Leica M165FC microscope (Leica, Wetzlar, Germany) for morphological assessment to determine number and sizes of lipid droplets.

NucBlue fluorescence data was used to correct the relative number of cells per well. First, the individual NucBlue RFU values of the control wells were averaged and set to a relative value of 1. Next, the RFU values of each treatment well was divided by the NucBlue control average. This created a ratio that was then used to normalize the AdipoRed data. Finally, the AdipoRed RFU values in each well were divided by its normalized NucBlue ratio. The average of each treatment group (n = 4) was calculated. When assessing changes in AdipoRed levels due to chemical exposure, the chemical profiles and individual chemicals were compared to the negative control.

### 2.6. Quantitative PCR of Adipogenic Transcripts

Following AdipoRed fluorescence measurements and morphological assessment, cells were lysed in Trizol (Thermo Fisher) and RNA was extracted for subsequent qPCR assessment. The purity and concentration of the RNA was measured using a NanoDrop One (Thermo Fisher). RNA (2 μg) was reverse transcribed into cDNA using MMLV-Reverse Transcriptase (Promega). Gene expression was measured on a BioRad iQ5 thermocycler (Hercules, CA, USA) using SYBR Green PCR master mix (BioRad) and gene-specific primers (Supplementary Table S1). All samples were run in triplicate. A cDNA standard was used to generate a standard curve with five concentration points consisting of 10^−3^ to 10^−7^ ng of cDNA to determine primer efficiency. To ensure there was an absence of non-specific primer binding, a melt curve was obtained for each run. β2-microglobulin (*β2mg*) and TATA binding protein (*Tbp*) were used as housekeeping genes to normalize the data, as previous studies demonstrate these transcripts are stable throughout adipocyte differentiation [34,35]. The ΔΔCT method [36] was used to analyze gene expression. Gene expression fold changes are compared to the gene expression of DMSO.

### 2.7. Pparγ Transactivation Assays

Transactivation plasmids were purchased from Addgene (Watertown, MA, USA). The reporter plasmid used was PPRE X3-TK-luc (Addgene plasmid #1015–Bruce Spiegelman), and the two expression plasmids used were pSG5 PPARα (Addgene plasmid #22751—Bruce Spiegelman) and PCS4 3XFLAG-PPARgamma1 (Addgene plasmid # 78769—Jaewhan Song) [37,38]. The pRL-SV40 Renilla plasmid (Promega, Madison, WI, USA) was used to normalize luciferase activity when a Dual-Glo assay was performed. DNA sequencing was performed by Psomagen (Rockville, MD, USA) to confirm plasmid sequencing and quality.

Transactivation assays were performed in HepG2 cells that were maintained under standard conditions (37 °C, 5% CO_2_) and fed with DMEM (Corning, Corning, NY, USA) containing 1% ITS (CorningA), 1% penicillin (Gibco, Waltham, MA, USA), and 10% fetal bovine serum (Corning). Either murine Pparα (NR1C1) or murine Pparγ (NR1C3) were transiently transfected along with the reporter plasmid into HepG2 cells using Effectene Reagent (Qiagen, Germantown, MD, USA) according to the manufacturer’s directions and as previously described [39]. Cells were plated at 1 × 10^5^ cells per well in 12-well plates and 24 h later exposed to chemical mixtures, individual environmental chemicals, or the positive controls rosiglitazone for PPARγ or GW590735 for PPARα. Steady Glo or Duo Glo luciferase assays were performed (Promega) according to the manufacturer’s directions 24 h after treatment (n = 6). DMSO was the solvent carrier for all chemicals, and so a DMSO negative control was performed.

### 2.8. Immunoblotting for Transcription Factors

An additional set of cells was differentiated during chemical exposure for 14 days, as described above. Cells were scraped into RIPA lysis buffer and centrifuged at 12,000 rpm to collect total protein. A final set of cells was differentiated for 7 days and used to separate nuclear and cytosolic proteins using the Nuclear and Cytoplasmic Extraction Reagents kit (Thermo Fisher). Protein concentrations were assessed by the BCA Assay (BioRad) and stored at −80 °C. Proteins (25 μg) were electrophoresed onto SDS polyacrylamide gels. Following transfer to nitrocellulose, primary antibodies for Pparg (SCBT, Santa Cruz, CA, USA, #SCBT7273, 1:200) and Ampk (Cell Signaling, Danvers, MA, USA, #2532S, 1:1000) were used, followed by HRP-labeled anti-mouse (SCBT) and anti-rabbit (GeneTex, Irvine, CA, USA) secondary antibodies. Protein expression was detected using Luminol (SCBT) with a BioRad ChemiDoc MP gel imager. β-actin (Cell Signaling, #8H10D10; 1:2000) was used as a loading control to normalize samples. Control expression levels were set to a value of 1 to calculate fold change in protein expression for the other groups.

### 2.9. Statistical Analysis

All data is presented as a mean ± SD. An ordinary one-way ANOVA with Tukey’s multiple comparisons test was used to determine statistical significance for lipid accumulation, cytotoxicity, protein expression, and gene expression. Datasets that had a significant difference between standard deviations used the Brown–Forsythe test and Welch ANOVA test (GraphPad Prism 10). Average lipid droplet size and count were analyzed in ImageJ (version 1.54h) by utilizing the tracing tool to trace lipid droplets. Linear regression analysis was performed in GraphPad.

## 3. Results

### 3.1. Mixture Profiles 2 and 5 Do Not Alter Adipogenesis

**Figure 1:**
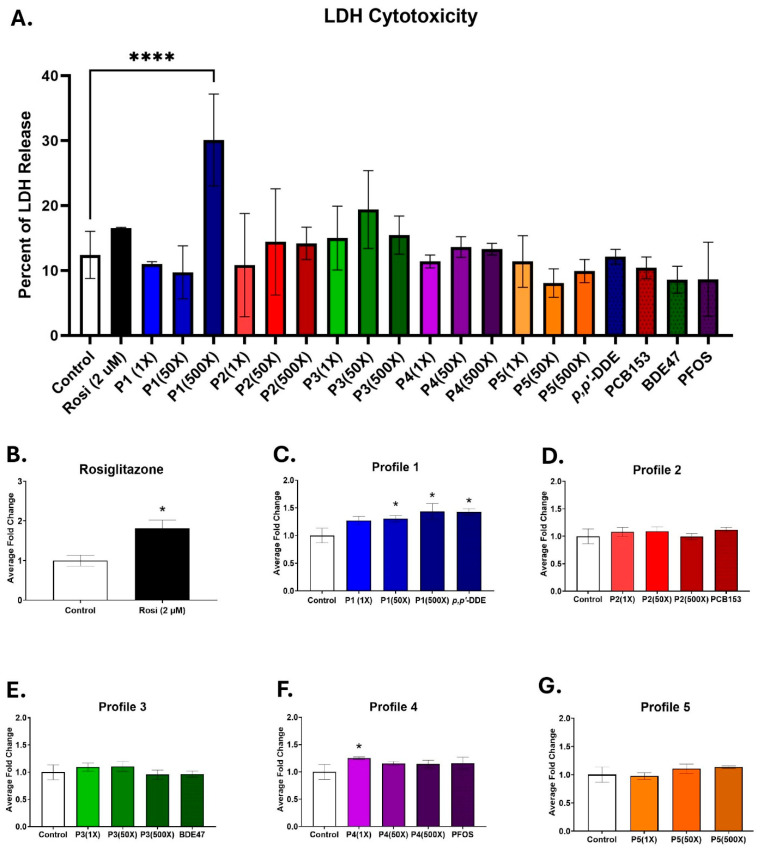
Profiles 1, 4, and *p*,*p*′-DDE exposures increase lipid accumulation. 3T3-L1 cells were exposed to DMSO (control), rosiglitazone (2 µM), Profiles 1–5 (1×, 50×, and 500×), or single chemicals *p*,*p*′-DDE, PCB153, BDE47, or PFOS (500×) for 14 days. Rosiglitazone (Rosi) served as the positive control. On D14, the cells were assessed for (**A**) LDH cytotoxicity and (**B**–**G**) triglyceride accumulation. Data was normalized to the control and is expressed as the average ± SD (n = 3–6 replicates per concentration per exposure group). Statistical differences were determined by a one-way ANOVA (* for *p* ≤ 0.05; **** for *p* ≤ 0.0001), followed by Tukey’s test.

3T3-L1 cells were exposed during a 14-day differentiation to one of the five different mixtures of *p*,*p*′-DDE, BDE47, PCB153, and PFOS, based upon measured serum levels in pregnant women (Table 1). Cells were cultured in three different concentrations of each mixture, at 1×, 50×, or 500× serum levels. To assess if exposure to the mixture behaved differently than the dominant chemical in each profile, cells were also exposed to 500× of the highest concentration of PCB153, BDE47, *p*,*p*′-DDE, or PFOS. Toxicity was assessed on day 14 by calculating the percentage of LDH released into the medium. None of the mixture profiles or individual chemicals exhibited any cytotoxic effects, except for Profile 1. At the 500× concentration, its LDH release into the medium was significantly elevated to 30.1%, compared to 12.4% in the control group (Figure 1A). No toxicity was observed at serum level (1×) concentrations.

Lipid accumulation in the differentiating 3T3-L1 cells was measured with AdipoRed, which binds to triglycerides. Rosiglitazone served as the positive control, since it is a known PPARγ agonist [40]. Triglyceride accumulation in the rosiglitazone-treated cells was 1.8-fold higher than the controls (Figure 1B). Triglyceride levels were also significantly elevated in the Profile 1 mixture, which has *p*,*p*′-DDE as its dominant chemical, by 1.3-, 1.3-, and 1.4-fold in the 1×, 50×, and 500× concentrations, respectively (Figure 1C). *p*,*p*′-DDE alone at 500× increased lipid accumulation by 1.4-fold, similar to that of the 500× Profile 1 (Figure 1C). Additionally, Profile 4 at the 1× concentration had lipid accumulation levels that were significantly increased by 1.3-fold, but no other concentration nor PFOS alone was changed (Figure 1F). No changes in lipid accumulation were noted for Profiles 2, 3, or 5 (Figure 1D,E,G).

Next, the expression of adipogenic marker genes was evaluated by qPCR. Alpha smooth muscle actin (*αSma*) is a marker for undifferentiated cells [41]. Peroxisome proliferator-activated receptor gamma (*Pparγ*) is a transcription factor that is known as the master regulator of adipogenesis [42]. Lipoprotein lipase (*Lpl*) is an extracellular enzyme that converts triglycerides (TGs) into fatty acids (FAs) [43]. These FAs are then brought into the adipocyte, where they will be bound by fatty acid-binding protein 4 (*Fabp4*) and either transported via carnitine palmitoyl transferase 1 (*Cpt1*) to the mitochondria to produce ATP via β-oxidation [44] or transported to lipid droplets to be stored [45]. Fat-specific protein 27 (*Fsp27*) is a protein that regulates lipolysis and triglyceride storage [46].

Using rosiglitazone as a positive control, mRNA levels of the six markers were examined by qPCR. Compared to the control cells, *αSma* was downregulated by 1.6-fold, while *Pparγ*, *Lpl*, *Fabp4*, *Fsp27*, and *Cpt1* were all significantly increased by 5.4-, 3.7-, 175.5-, 32.3-, and 4.4-fold, respectively (Figure S1).

Adipogenic changes after exposure to mixture Profiles 2 and 5 were examined next. Exposing 3T3-L1 cells to the dominant chemical in Profile 2, PCB153, did not alter adipogenic mRNA expression (Figure S2). However, lower concentrations of the Profile 2 mixture did increase the adipogenic genes *Ppary*, *Fabp4*, and *Fsp27* by 1.7-, 4.6-, and 7.9-fold at the 50× concentration (Figure S2). Exposure of 3T3-L1 cells to Profile 5 resulted in no increased triglyceride accumulation, nor any changes to adipogenic marker transcripts (Figure S3).

### 3.2. Profile 1 and p,p′-DDE Exposure Result in Similar Gene Expression Patterns

**Figure 2:**
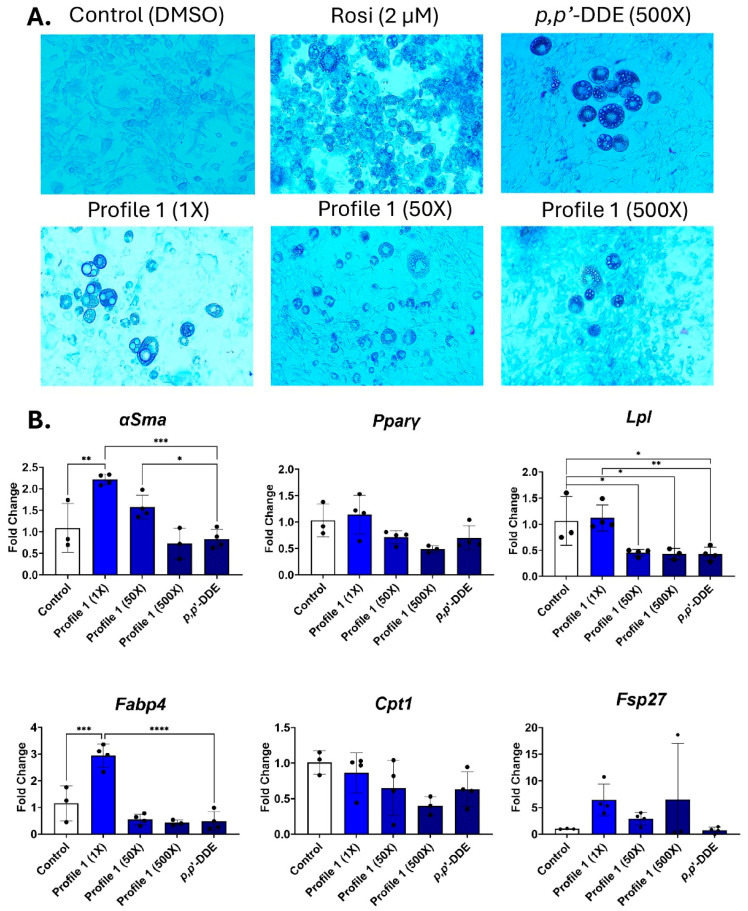
Profile 1 and *p*,*p*′-DDE exposures decrease *Lpl* expression and increase lipid accumulation. 3T3-L1 cells were exposed to DMSO (control), Rosi (2 µM), Profile 1 (1×, 50×, and 500×), or single chemical *p*,*p*′-DDE (500×) for 14 days. (**A**) On day 14, morphological changes of 3T3-L1 cells were evaluated at 100X magnification and representative brightfield images are presented above. (**B**) Transcript levels of *αSma* (fibroblast marker), *Pparγ* (transcription factor), *Lpl* (extracellular enzyme), *Fabp4* (fatty acid-binding protein), *Cpt1* (mitochondrial transporter), and *Fsp27* (lipid droplet associated protein) were also measured on day 14. β2-microglobulin (*β2mg*) and TATA binding protein (*Tbp*) were used as housekeeping genes to normalize the data. The ΔΔCT method was used to analyze gene expression (n = 3–6 replicates per concentration per exposure group). Statistical differences were determined by a one-way ANOVA followed by Tukey’s multiple comparisons test (* for *p* ≤ 0.05; ** for *p* ≤ 0.01; *** for *p* ≤ 0.001; **** for *p* ≤ 0.0001).

The day 14 differentiated 3T3-L1 cells were imaged to view morphological changes. In all cases, the DMSO-treated cells remain as fibroblasts, while the rosiglitazone-treated cells appear to be mature adipocytes with rounded cells that contain differing sizes of lipid droplets (Figure 2A). The single exposure of 500× *p*,*p*′-DDE and the 500× concentration of Profile 1 have lower numbers of cells containing lipid droplets, while the 1× concentration has large lipid droplets that take up a majority of the adipocyte (Figure 2A and Figure S4).

In mixture Profile 1, both *αSma* and *Fabp4* transcripts were increased at the 1× concentration by 2.2- and 2.9-fold, respectively (Figure 2B). However, these changes were not seen in the two higher concentrations, nor in the *p*,*p*′-DDE exposure. *Lpl* expression is reduced by 2.2- and 2.3-fold in the 50× and 500× concentrations, respectively. *p*,*p*′-DDE is the dominant chemical in Profile 1, and its individual 500× exposure also downregulated *Lpl* by 2.4-fold (Figure 2B).

### 3.3. Mixture Profile 4 and Its Dominant Chemical PFOS Express Similar Adipogenic Transcripts

**Figure 3:**
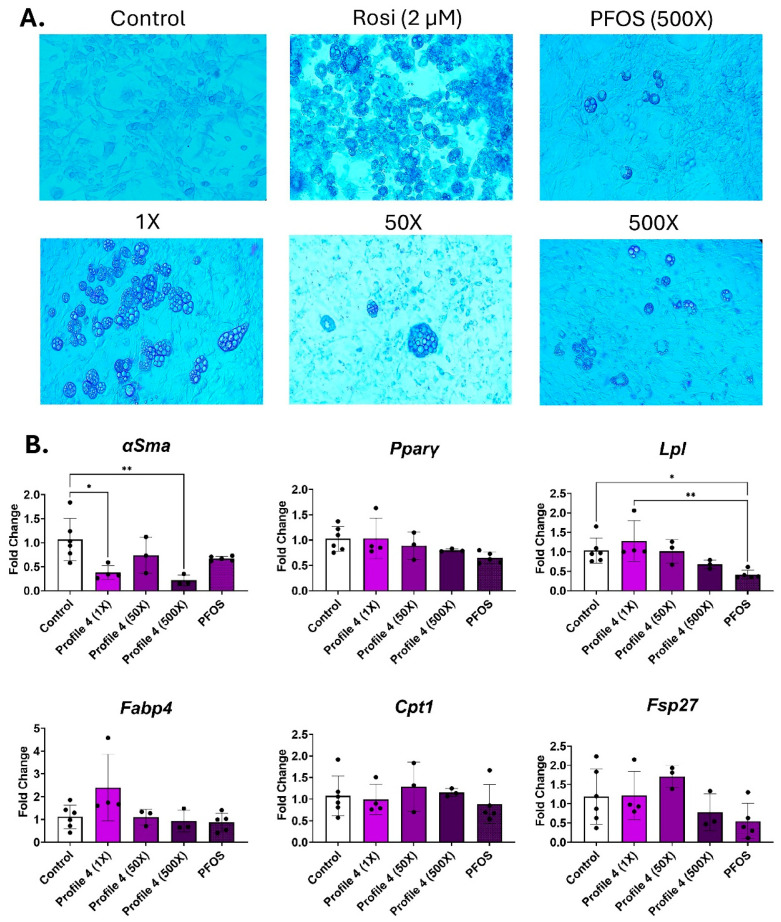
Profile 4 and PFOS exposure decreases *Lpl* expression and increases lipid accumulation. 3T3-L1 cells were exposed to DMSO (control), Rosi (2 µM), Profile 4 (1×, 50×, and 500×), or single chemical PFOS (500×) for 14 days. (**A**) On day 14, morphological changes of 3T3-L1 cells were evaluated at 100X magnification and representative brightfield images are presented above. (**B**) Transcript levels of *αSma* (fibroblast marker), *Pparγ* (transcription factor), *Lpl* (extracellular enzyme), *Fabp4* (fatty acid-binding protein), *Cpt1* (mitochondrial transporter), and *Fsp27* (lipid droplet associated protein) were also measured on day 14. β2-microglobulin (*β2mg*) and TATA binding protein (*Tbp*) were used as housekeeping genes to normalize the data. The ΔΔCT method was used to analyze gene expression (n = 3–6 replicates per concentration per exposure group). Statistical differences were determined by a one-way ANOVA followed by Tukey’s multiple comparisons test (* for *p* ≤ 0.05; ** for *p* ≤ 0.01).

In mixture Profile 4, the predominant chemical is PFOS. Following 14 days of differentiation, cell morphology was examined. Lipid droplets were seen in all exposures, but only the 1× and 500× groups had large numbers of lipid droplets, while the 50× concentration of Profile 4 had lower numbers of droplets, but they were large sized (Figure 3A and Figure S5A,B). The 500× PFOS-only exposure group had droplet sizes that were similar to those of the 500× mixture; however, the 500× Profile 4 mixture had higher numbers of droplets compared to PFOS alone (Figure 3A and Figure S5A,B). *αSma*, the marker of undifferentiated cells, was reduced by 3-fold in the 1× and 500× concentrations (Figure 3B). The only other notable change in expression patterns was the dose-dependent downregulation of *Lpl* expression in Profile 4, which is concurrent with 2.7-fold lower levels of *Lpl* in the 500× PFOS-only exposure group (Figure 3B).

### 3.4. Differential Transcript Expression Between Mixture Profile 3 and BDE47

**Figure 4:**
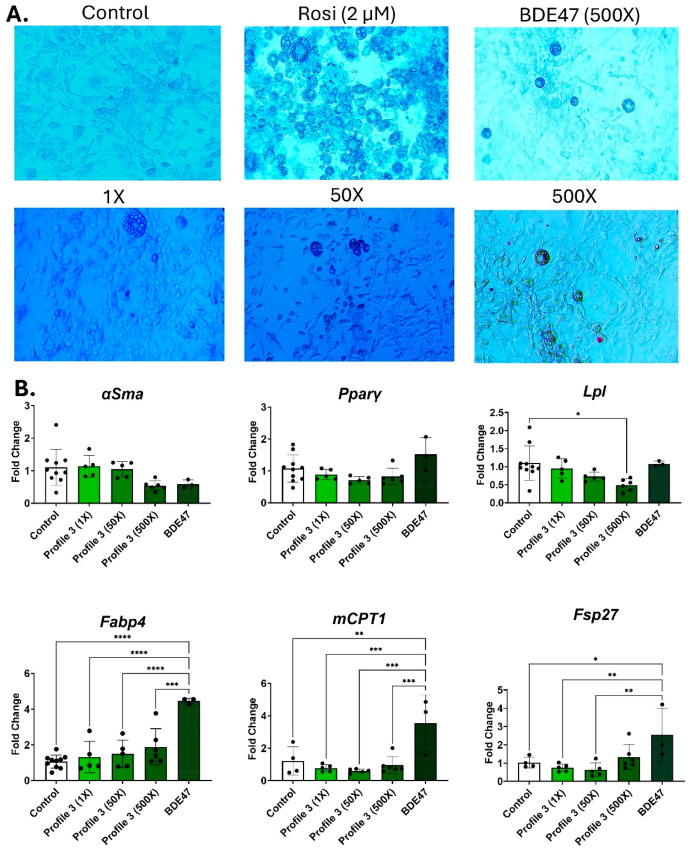
BDE47 exposure decreases *Lpl*, *Fabp4*, *and Cpt1* expression, but causes no change to lipid accumulation. 3T3-L1 cells were exposed to DMSO (control), Rosi (2 µM), Profile 3 (1×, 50×, and 500×), or single chemical BDE47 (500×) for 14 days. (**A**) On day 14, morphological changes of 3T3-L1 cells were evaluated at 100X magnification and representative brightfield images are presented above. (**B**) Transcript levels of *αSma* (fibroblast marker), *Pparγ* (transcription factor), *Lpl* (extracellular enzyme), *Fabp4* (fatty acid-binding protein), *Cpt1* (mitochondrial transporter), and *Fsp27* (lipid droplet associated protein) were also measured on day 14. β2-microglobulin (*β2mg*) and TATA binding protein (*Tbp*) were used as housekeeping genes to normalize the data. The ΔΔCT method was used to analyze gene expression (n = 3–6 replicates per concentration per exposure group). Statistical differences were determined by a one-way ANOVA followed by Tukey’s multiple comparisons test (* for *p* ≤ 0.05; ** for *p* ≤ 0.01; *** for *p* ≤ 0.001; **** for *p* ≤ 0.0001).

BDE47 is the dominant chemical in mixture Profile 3. After 14 days of adipogenic differentiation, BDE47 alone at 500× concentration induced a high number of small lipid droplets (Figure 4A and Figure S6A). In contrast, the 500× Profile 3 had few lipid droplets present, but these were large (Figure 4A and Figure S6B). Similarly, the cells exposed to mixture Profile 3 showed patterns of adipogenic transcript markers that were different from its dominant chemical BDE47. In the 500× BDE47 exposure groups, *Lpl* expression was dose-dependently reduced in the Profile 3 exposures, while remaining at control levels in the 500× BDE47-only exposure group (Figure 4B). In contrast, *Fabp4*, *Cpt1*, and *Fsp27* were all significantly elevated by 4.5-, 2.6-, and 3.5-fold, respectively, in the 500× BDE47 exposure group, while there were no significant changes in these markers in the Profile 3 mixture exposure groups (Figure 4B).

### 3.5. Changes in Nuclear Pparg and Ampk Protein Expression

**Figure 5:**
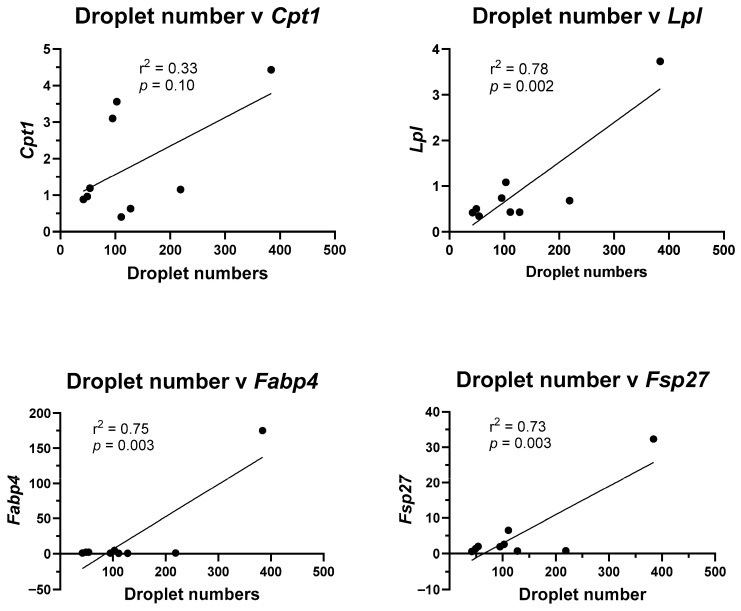
Lipid droplet numbers correlate with adipogenic transcript levels. Lipid droplet numbers in the control, rosiglitazone, and 500× concentration of each individual chemical and 500× concentration of Profiles 1–4 were plotted against mRNA expression of the adipogenic transcripts. Linear regression (GraphPad Prism) was used to determine significant correlations (*p* ≤ 0.05).

To determine potential mechanisms for the changes in lipid droplet formation and adipogenic transcript marker expression, correlations between the two were examined. Using linear regression, the average number of lipid droplets in the control, rosiglitazone, and 500× concentrations of mixture Profiles 1, 2, 3, and 4, and the 500× concentration of *p*,*p*′-DDE, PCB153, BDE47, and PFOS, were plotted against transcript levels. Although there was a trend, no significant correlation was found between lipid droplet numbers and *Cpt1* levels (Figure 5). In contrast, lipid droplet numbers are significantly correlated with *Lpl* levels (*p* = 0.002), the enzyme that converts triglycerides into fatty acids, with *Fabp4* levels (*p* = 0.003), a fatty acid cellular binding protein, and *Fsp27* levels (*p* = 0.003), a protein that regulates lipolysis (Figure 5).

**Figure 6:**
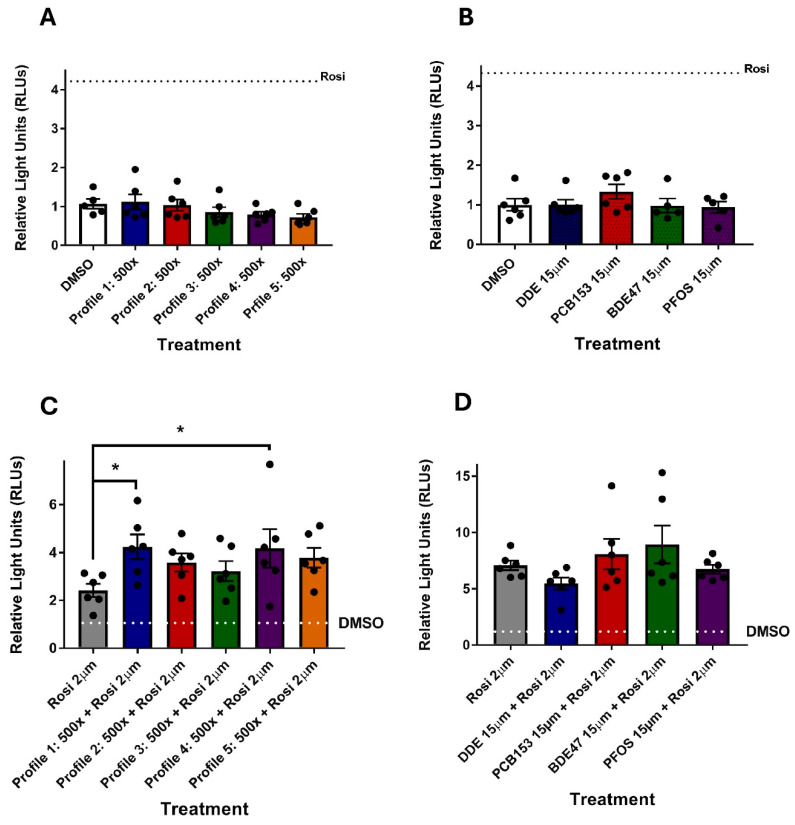
Exposure to profile mixtures at 500× and individual chemicals at 15 μM does not activate PPARγ. PPARγ transactivation assays were performed using rosiglitazone (Rosi) as the positive control. (**A**) Profiles 1–5 (500×) and (**B**) individual chemicals at 15 μM were assessed for PPARγ transactivation activity. To assess antagonistic effects, (**C**) rosiglitazone (2 μM) was co-incubated with Profiles 1–5 (500×) and (**D**) individual chemicals at 15 μM. Each sample was then assessed for PPARγ transactivation activity. Statistical differences (*) were determined by a one-way ANOVA followed by Fisher’s LSD test (*p* ≤ 0.05; n = 6).

We hypothesized that the activation of Pparγ, a key transcription factor important for adipogenesis [47], would be altered during exposure to either individual chemical or the profile mixtures. However, none of the mixture profiles activated Pparγ after a 24 h exposure (Figure 6A). Neither did any of the individual chemicals (Figure 6B). Therefore, we investigated whether the mixtures or individual chemicals acted as antagonists to the Pparγ activator rosiglitazone. Surprisingly, none of the profile mixtures or individual chemicals acted as antagonists (Figure 6C,D); instead, Profiles 1 and 4 significantly enhanced rosiglitazone-mediated PPARγ activity. It appears as if rosiglitazone is necessary to prime the Pparγ activity of chemicals similar to several obesogens where a high-fat diet is necessary for the measured chemically mediated obesogenic response [48,49,50].

**Figure 7:**
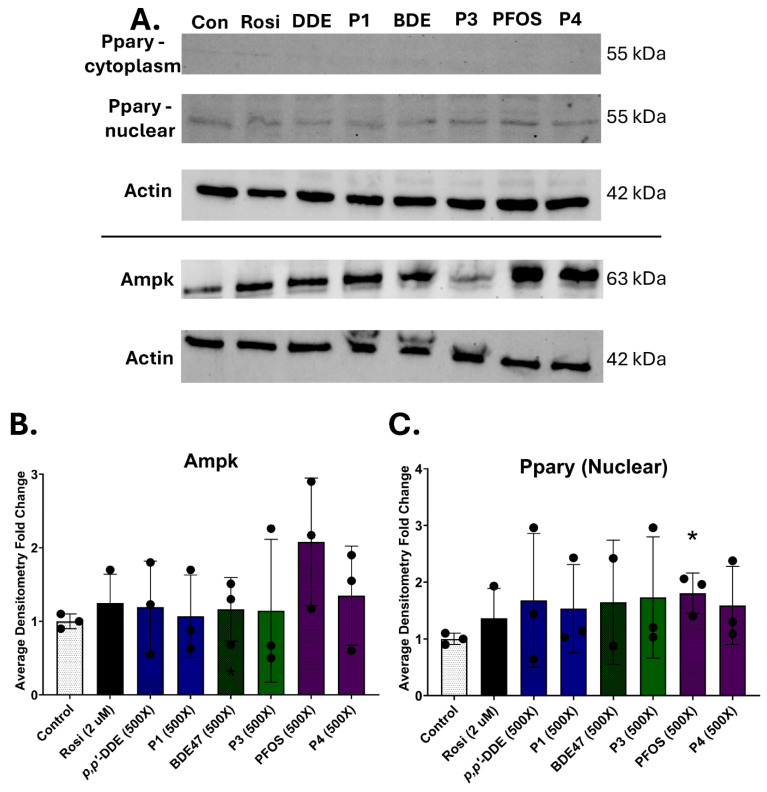
Protein levels of Pparγ and Ampk. Profiles 1, 3, and 4, and their dominant chemicals (*p*,*p*′-DDE, BDE47, and PFOS) were (**A**) examined for changes in Pparγ or Ampk protein expression by immunoblotting. b-actin was used as a loading control. (**B**,**C**) Densitometry values of Pparγ and Ampk replicates (n = 3) were averaged and normalized to β-actin. The control samples were set to a relative value of 1 to calculate fold-change in expression for the exposure groups. Statistical differences (*) were determined by a one-way ANOVA followed by Tukey’s multiple comparisons test (*p* ≤ 0.05).

Since the transactivation assays were conducted for a short time period, we wondered if protein levels of Pparγ would be changed during the differentiation process. 3T3-L1 cells were exposed for 7 days to Profiles 1, 3, and 4, and their dominant chemicals, *p*,*p*′-DDE, BDE47, and PFOS. Little to no Pparγ protein was detected in the cytoplasm (Figure 7A). Nuclear Pparγ protein levels were elevated in all samples, but only PFOS at the 500× concentration had a statistically significant increase of 1.8-fold in comparison to the control (Figure 7B). Adenosine monophosphate-activated protein kinase (Ampk) is another key protein during adipogenic differentiation. Ampk inhibits adipogenesis by reducing the function of both C/EBPα and PPARγ [20]. Investigating Ampk expression after 14 days of differentiation revealed a significant 2-fold induction in Ampk protein levels in the PFOS exposure group (Figure 7A,C).

**Figure 8:**
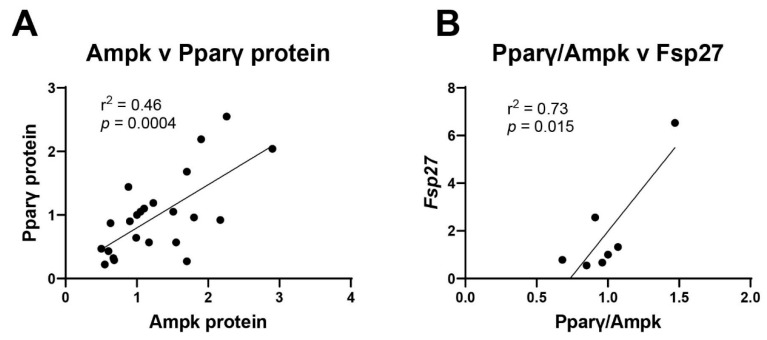
Multiple transcription factors work in concert to alter adipogenesis. (**A**) Pparγ and Ampk protein expression values were plotted and their correlation determined by linear regression. (**B**) The ratio of Pparγ to Ampk was plotted against mRNA expression of *Fsp27*. Linear regression (GraphPad Prism) was used to determine signficant correlations (*p* ≤ 0.05).

Next, the relationship between Pparγ and Ampk was explored. There is a highly significant correlation (*p* = 0.0004) between expression of Pparγ protein levels on day 7 and Ampk protein levels on day 14 (Figure 8A). Expressing this relationship as a ratio to hone in on how transcription factors interplay with each other, linear regression analysis shows that the ratio of Pparγ to Ampk is significantly associated with *Fsp27* levels (Figure 8B). In general, increased relative amounts of Pparγ results in increased *Fsp27* levels, while higher Ampk results in reduced *Fsp27* levels (Figure 8B). These findings suggest that each individual chemical uniquely induces or represses transcription factors that control adipogenesis. When the chemicals are combined in mixtures, it is the mechanistic interplay between transcription factors that are responsible for synergistic or antagonistic chemical interactions.

## 4. Discussion

The results of this study indicate that real-world chemical mixtures can alter adipocyte differentiation, lipid droplet formation, and adipogenic marker gene expression differently compared to single chemical exposures. For example, cell exposure to mixture Profile 3 resulted in differential expression of *Fsp27*, *Fabp4*, and *Lpl*, compared to the dominant chemical in Profile 3, BDE47. In contrast, high levels of PFOS appear to downregulate some, but not all, adipogenic markers. These findings indicate that investigating chemical mixtures that mirror serum concentrations is important to understand the drivers of adipogenesis.

### 4.1. Profile 3 Increases Adipogenesis in Comparison to BDE47 Alone

The dominant chemical in serum Profile 3 is BDE47. Several other studies have shown that BDE47 exposure can alter adipogenesis in 3T3-L1 cells. For example, concentrations of BDE47 between 2.5 and 10 μM increased both triglyceride levels and expression of the adipogenic genes Leptin (*Lep*) and *Pparγ* [24]. In our study, the 500× concentration of BDE47 was even lower, at 0.95 μM, but still altered adipogenic gene expression. 3T3-L1 cells exposed to a mixture of PBDEs or to BDE47 alone had increased Perilipin protein expression at 12.8 μM and 25 μM of the PBDE mixture, and increased Fabp4 protein expression at 25 μM of the PBDE mixture [51]. However, both Fabp4 and Perilipin were increased when exposed to 6 μM BDE47, which is a lower concentration of BDE47 than within the mixture [51]. These findings align with our data in which 500× BDE47 alone (or 0.95 μM BDE47) significantly elevated transcript levels of *Cpt1*, *Fsp27*, and *Fabp4* by 3.5-, 2.6, and 4.5- fold, respectively. However, expression of the adipocyte formation markers is not different from control cells when BDE47 is combined with three other chemicals (PFOS, PCB153, and *p*,*p*′-DDE) to comprise 500× of the serum Profile 3 mixture. These results suggest that the other chemicals in Profile 3 are attenuating or have an antagonistic effect on BDE47.

There are three transcripts increased by BDE47 in the current study: *Cpt1*, *Fsp27*, and *Fabp4*. Fabp4 binds fatty acids (FAs) entering the cell, which can then be transported either to the mitochondria for β-oxidation or to lipid droplets for storage [52]. Cpt1 transports long-chain fatty acids into mitochondria, which use these FAs to generate ATP for the cell [53]. Under normal conditions, an increase in β-oxidation within the cell promotes “browning” of the adipocyte, which creates small, multilocular lipid droplets [54]. The cells exposed to only BDE47 in this study have similarly increased lipid droplet numbers, while the droplets themselves were small in size. Indeed, another study that exposed 3T3-L1 cells to 5 or 10 μM of BDE47 saw an increase in both mitochondrial respiration and lipid accumulation [55], which is concurrent with the observed increase in both *Cpt1* and *Fsp27* in the current study.

In searching for a reason for the differential changes between BDE47 alone and in a mixture, we first conducted a Pparγ transactivation assay. However, the 24 h Pparγ transactivation assays showed no differences between the control and exposure to individual chemicals or to mixtures. We next tested whether the chemical mixture profiles altered PPARα activity. Interestingly, only Profile 3 significantly activated PPARα transactivation activity (Figure S8A). Further, BDE47, the dominant chemical in Profile 3, and PCB153 were activators of PPARα at 15 μM (Figure S8B). However, at the concentrations found in Profile 3 (see Table 1), neither BDE47 nor PCB153 were sufficient to activate PPARα. This suggests that the individual compounds within the mixture alone were not sufficient to act as obesogens through PPARα, but could do so when combined as a mixture. Transactivation activity was increased by the addition of each chemical: BDE47 < BDE47 + PCB153 < chemical Profile 3 (Figure S8C). Profile 3 also contains PFOS, which has been shown to activate PPARα in vivo [56,57], and to activate PPARα in transactivation assays, but only at 100 μM PFOS or greater [18,58,59].

Most studies examining the mechanisms of chemical-induced adipogenesis rely on assays to test the chemical binding and/or transactivation of Ppar family members, which are short-term assays. While PPARγ and possibly PPARα activation may be part of the mechanism for the differential obesogenic activity of these chemical mixture profiles, they do not provide the only mechanism. In the current study, the activation of each receptor alone was not strong, and high concentrations were needed. We therefore next examined Pparγ nuclear translocation and protein expression after 7 days of exposure. Immunoblots that show low to no levels of cytoplasmic Pparγ, but robust expression of nuclear Pparγ, indicate that most of the protein is being translocated to the nucleus. While the data indicates an increase in nuclear Pparγ protein in Profile 3 and BDE47 at the 500× concentration, this was not statistically significant.

Though Pparγ is considered the master regulator of adipogenesis, there are several other transcription factors that also contribute to adipocyte formation and maturation. One of the proteins is Ampk, which alters the function of both Pparγ and C/ebpα [19]. Therefore, Ampk protein levels were assessed. While Ampk expression appears to be reduced in the Profile 3 exposure, it was also not statistically different. However, the combined changes in expression between the two transcription factors helps explain the differences in adipogenic transcripts, such as *Fsp27*, and lipid droplet formation. The BDE47 exposure group has increased nuclear Pparγ along with reduced Ampk protein levels, which suggest a robust increase in adipogenesis. The Profile 3 exposure group has only an increased nuclear Pparγ, without changes in Ampk, which suggests a more muted adipogenic response. Indeed, linear regression reveals that expression of these two proteins are correlated. Further, evaluating the Pparγ/Ampk ratio against adipogenic transcripts also reveals a positive correlation with *Fsp27*, a protein that regulates lipolysis and triglyceride storage [46].

### 4.2. Profile 4 and PFOS Reduce Lpl Expression

The dominant chemical in Profile 4 is PFOS. PFOS alone at 500× serum concentration reduces *Lpl* transcript expression, an extracellular enzyme that converts circulating TGs into fatty acids [60]. In contrast, the 1× serum concentration of Profile 4 increases *Lpl* expression. As the concentration of Profile 4 increases to 50× and 500× serum levels, *Lpl* expression is reduced to control levels in a dose-dependent manner. High *Lpl* levels mean that there would be increased amounts of FAs entering the adipocyte. These findings strongly correlate with the morphological data, in which the 1× mixture had the highest number of lipid droplets compared to the other Profile 4 exposure groups.

In the literature, there are conflicting results with PFOS exposure during 3T3-L1 cell differentiation. Studies have found that concentrations of PFOS between 1 and 300 μM increased triglyceride accumulation, while concentrations of 50 μM PFOS increased expression of *Fabp4*, *Lpl*, and *Pparγ* at day 3 of differentiation [27,61]. ln our study, the 500× PFOS concentration is equivalent to 10.8 μM. Conversely, another study found that PFOS alone did not cause an increase in lipid accumulation, but a mixture of PFAS increased lipid accumulation at 10 and 100 nM [62]. The PFOS concentration at 1× mixture Profile 4 is 21.5 nM. Our findings of an increase in adipogenic genes at relatively low concentrations of PFOS, but no changes at higher concentrations of PFOS, are in accordance with these two studies. This implies that PFOS has a U-shaped dose–response curve for adipogenesis.

To assess mechanisms, other groups have used Pparγ transactivation studies to show that 1 μM PFOS or 10 μM of PFAS mixture do not act as agonists, but rather had antagonistic effects [62]. Our 24 h transactivation assays indicate that a higher concentration of 15 μM PFOS does not activate or antagonize Pparγ. While 500× of Profile 4 (containing 10.8 μM PFOS) does not activate Pparγ alone, it can enhance the effects of rosiglitazone, a known Pparγ agonist. However, when cells are exposed to PFOS for longer durations, nuclear translocation of Pparγ is altered. In the current study, a 7-day exposure to mixture profiles and individual chemicals demonstrated an elevation in nuclear Pparγ protein levels in all samples, but only PFOS at the 500× concentration significantly increased nuclear Pparγ protein by 1.8-fold. Interestingly, 500× PFOS also significantly induced Ampk protein levels by 2-fold. We hypothesize that the changes in adipogenesis at low concentrations may be due to preferential binding to these two transcription factors. It may be that, at low nM (1×) concentrations of PFOS, there is Pparγ nuclear translocation only, while at higher PFOS concentrations, both Ampk and Pparγ translocate to the nucleus, thus dampening each other’s effect.

This study lays the groundwork to help better understand the link between maternal exposures and the effects these mixtures have on metabolic or obesity outcomes in children. It is known that exposure to chemicals during fetal development, when adipocyte development primarily occurs, can change the numbers and/or function of adipocytes [9,63]. Indeed, several human studies have linked maternal serum chemical levels with increases in childhood BMI [12,13]. However, the mechanisms that drive changes in adipocytes, ultimately manifesting as increased BMI, remain unknown. Thus, it is important to assess real-life chemical mixtures and generate models that integrate effects on multiple molecular target molecules to address the increases in childhood obesity.

## 5. Conclusions

With over 350,000 chemicals being registered for production use [1], it is important to assess the effects of real-world chemical mixtures. Profile 1, with the dominant chemical of *p*,*p*′-DDE, did not alter adipogenic differentiation of 3T3-L1 cells. Profile 4, with the dominant chemical of PFOS, appeared to have a slight mixture effect, and Profile 3, with the dominant chemical of BDE47, appeared to have a strong antagonistic effect on 3T3-L1 cell differentiation, as compared to BDE47 alone. This research has allowed us to streamline the approaches to identifying and investigating the toxic effects of real-world-exposure to chemical mixtures and will propel our efforts forward into determining the mechanism behind these mixture effects.

## Figures and Tables

**Table 1:** Average serum concentrations of chemicals in each mixture profile at 1× (nM).

Mixture Profile #	*p*,*p*′-DDE	BDE47	PCB153	PFOS
**1**	28.4	0.2	0.1	9.3
**2**	3.9	0.1	0.5	17.6
**3**	2.0	1.9	0.1	13.6
**4**	1.4	0.2	0.1	21.5
**5**	1.5	0.1	0.1	7.6

## Data Availability

The original contributions presented in this study are included in the article/Supplementary Materials. Further enquiries can be directed to the corresponding author.

## Supplementary Materials

The following supporting information can be downloaded at: https://www.mdpi.com/article/10.3390/toxics13110975/s1: Table S1. Primer sequences used in qPCR; Figure S1. Rosiglitazone exposure reduces *αSma* expression but increases *Pparγ*, *Lpl*, *Fabp4*, *Fsp27*, and *Cpt1* expression; Figure S2. Profile 2 modestly increases *αSma*, *Pparγ*, *Fabp4*, and *Fsp27* expression. Figure S3. Profile 5 exposure does not alter adipogenic transcript expression; Figure S4. Profile 1 at the 500× concentration and *p*,*p*′-DDE at the 500× concentration have similar lipid droplet numbers and sizes; Figure S5. Profile 4 at the 500× concentration has higher numbers of lipid droplets in comparison to PFOS at the 500× concentration, but similar sizes of lipid droplets; Figure S6. Profile 3 at the 500× concentration and BDE47 at the 500× concentration have different numbers and sizes of lipid droplets; Figure S7. Profile 2 at the 500× concentration and PCB153 at the 500× concentration have the same number of lipid droplets, but different lipid sizes; Figure S8. Exposure to Profile mixture 3 at 500×, PCB153, and BDE47 individually at 15 μM activates PPARα in HepG2 cells.
