# Supplementary material for: Mixtures of p,p′-DDE, PCB153, BDE47, and PFOS Alter Adipocytic Differentiation of 3T3-L1 Cells
Source: Toxics. 2025 Nov 13;13(11):975. doi: 10.3390/toxics13110975 (PMC12656446; doi:10.3390/toxics13110975)

Supplementary Materials

Table S1: Primer sequences used in qPCR			
**Gene**	Function	Sequences (5'-3')	Annealing Temp (°C)
β2mg	Housekeeping gene	F: 5'-GGTCTTTCTGGTGCTTGTCT-3'<br>R: 5'-TATGTTCGGCTTCCCATTCTC-3'	54
Tbp	Housekeeping gene	F: 5'-CTACCGTGAATCTTGGCTGTA-3'<br>R: 5'-GTTGTCCGTGGCTCTCTTATT-3'	58
αSma	Fibroblast marker	F: 5'-TCAGGGAGTAATGGTTGGAATG-3'<br>R: 5'-GGTGATGATGCCGTGTTCTA-3'	60
Fabp4	Protein that regulates the metabolism of fatty acids in adipocytes	F: 5'-GTGAAGAGCATCATAACCCTAGAT-3'<br>R: 5'-CACGCCTTTCATAACACATTC-3'	58
Pparγ	Transcription factor that regulates lipid, glucose, and amino acid metabolism	F: 5'-CCCTGGCAAAGCATTTGTATG-3'<br>R: 5'-GGTGATTTGTCCGTTGTCTTTC-3'	58
Lpl	Extracellular enzyme that converts triglycerides to fatty acids	F: 5'-AGCAGGAAGTCTGACCAATAAG-3'<br>R: 5'-ATCAGCGTCATCAGGAGAAAG-3'	55
Cpt1	Protein transports long chain fatty acids into the mitochondria for beta oxidation	F: 5'-ATTCTGTGCGGCCCTTATT-3'<br>R: 5'-TGACTTGAGCACCAGGTATTT-3'	58
Fsp27	Protein that regulates lipolysis and triglyceride storage	F: 5'-GCTGAACCCTCAGGACTTTATT-3'<br>R: 5'-CTTGTAGCAGTGCAGGTCATA-3'	58

Figure S1. Rosiglitazone exposure reduces αSma expression but increases *Pparγ*, *Lpl, Fabp4, Fsp27 and Cpt1* expression, 3T3-L1 cells were exposed to DMSO (control) or Rosi (2 μM) for 14 days. On day 14, transcript levels of *αSma* (fibroblast marker), *Pparγ* (transcription factor), *Lpl* (extracellular enzyme), *Fabp4* (fatty acid binding protein), *Cpt1* (mitochondrial transporter), and *Fsp27* (lipid droplet associated protein) were measured. β2-Microglobulin (*β2mg*) and TATA binding protein (*Tbp*) were used as housekeeping genes to normalize the data. The ΔΔCT method was used to analyze gene expression (n = 16-20 replicates per gene). Statistical differences (*) were determined by Student's t-test ($p \leq 0.05$).

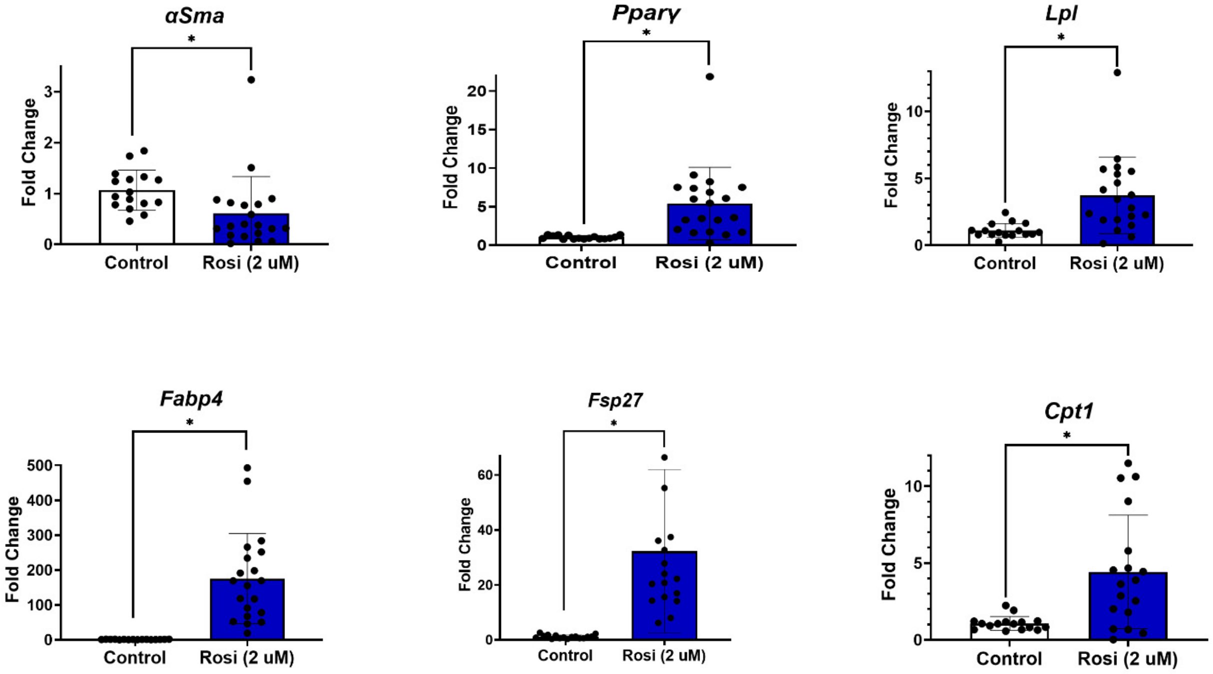

Figure S2. Profile 2 modestly increases *αSma*, *Pparγ*, *Fabp4*, and *Fsp27* expression. 3T3-L1 cells were exposed to DMSO (control), Rosi (2 μM), Profile 2 (1X, 50X, and 500X), or single chemicals PCB153 (500x) for 14 days. On day 14, transcript levels of *αSMA* (fibroblast marker), *Pparγ* (transcription factor), *Lpl* (extracellular enzyme), *Fabp4* (fatty acid binding protein), *Cpt1* (mitochondrial transporter), and *Fsp27* (lipid droplet associated protein) were measured. β2-Microglobulin (*β2mg*) and TATA binding protein (*Tbp*) were used as housekeeping genes to normalize the data. The ΔΔCT method was used to analyze gene expression (n=3-6 replicates per concentration per exposure group. Statistical differences were determined by a one-way ANOVA (* for $p \leq 0.05$; **** for $p \leq 0.0001$), followed by Tukey's test.

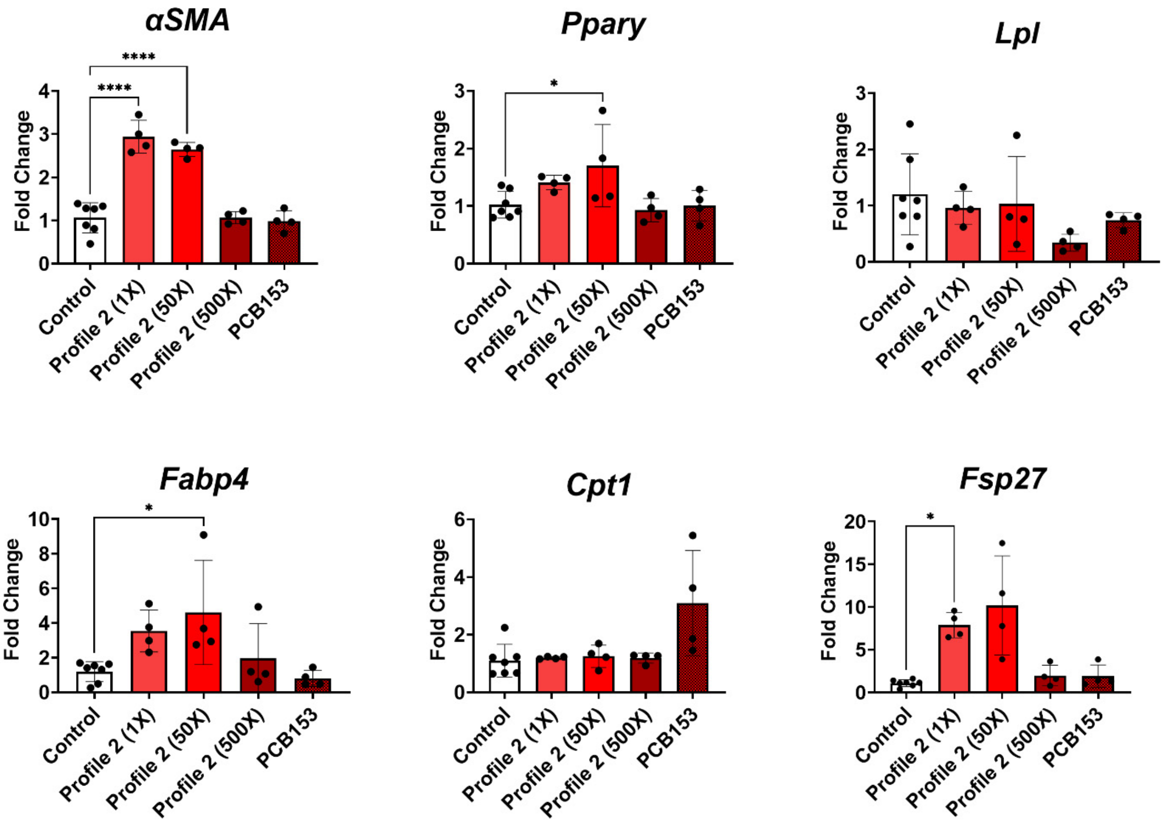

Figure S3. Profile 5 exposure does not alter adipogenic transcript expression. 3T3-L1 cells were exposed to DMSO (control), Rosi (2 µM), Profiles 5 (1X, 50X, and 500X) for 14 days. On day 14, transcript levels of *αSMA* (fibroblast marker), *Pparγ* (transcription factor), *Lpl* (extracellular enzyme), *Fabp4* (fatty acid binding protein), *Cpt1* (mitochondrial transporter), and *Fsp27* (lipid droplet associated protein) were measured. β2-Microglobulin (*β2mg*) and TATA binding protein (*Tbp*) were used as housekeeping genes to normalize the data. The ΔΔCT method was used to analyze gene expression (n=3-6 replicates per concentration per exposure group.

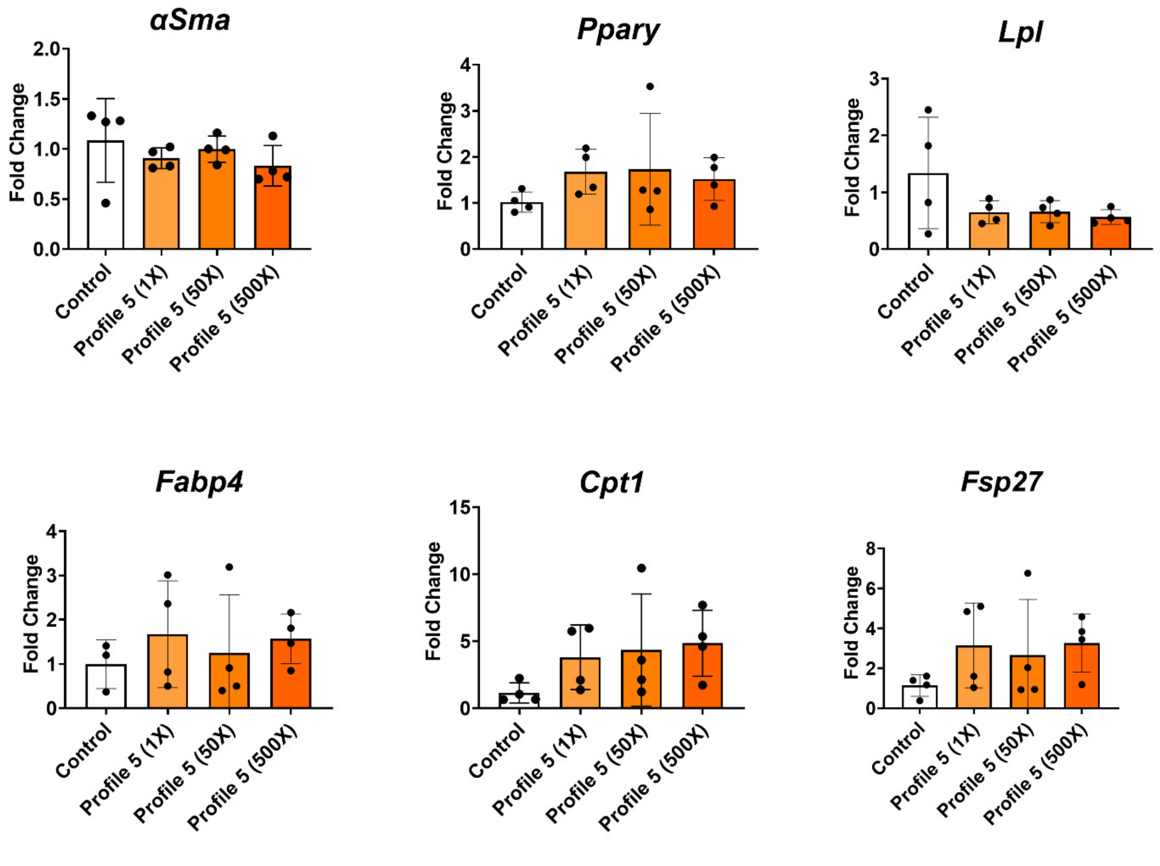

Figure S4. Profile 1 at the 500X concentration and *p,p'*-DDE at the 500X concentration have similar lipid droplet numbers and sizes. Brightfield images of 3T3-L1 cells were evaluated for A) number of droplets, and B) average droplet size via ImageJ after exposure to Profile 1 at the 1X, 50X, and 500X concentrations as well as a single exposure of *p,p'*-DDE (500X).

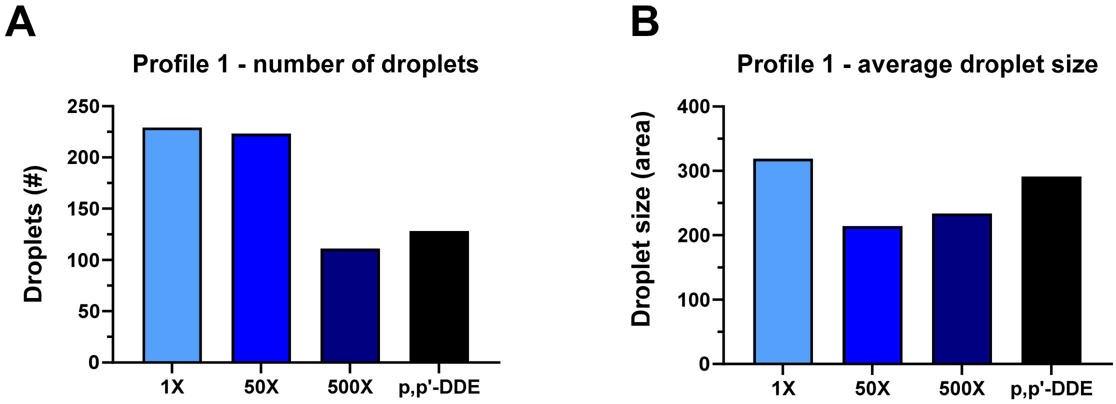

Figure S5. Profile 4 at the 500X concentration has higher numbers of lipid droplets in comparison to PFOS at the 500X concentration, but similar sizes of lipid droplets. Brightfield images of 3T3-L1 cells were evaluated for A) number of droplets, and B) average droplet size via ImageJ after exposure to Profile 4 at the 1X, 50X, and 500X concentrations as well as a single exposure of PFOS (500X).

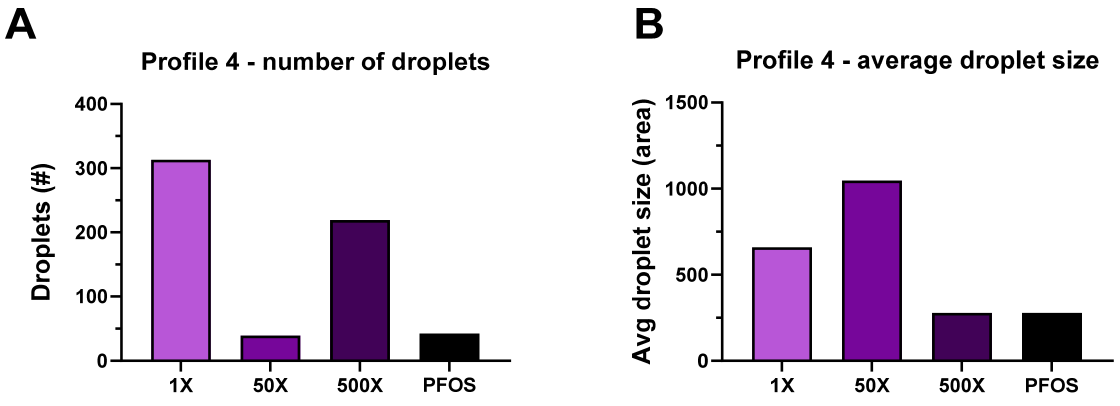

Figure S6. Profile 3 at the 500X concentration and BDE47 at the 500X concentration have different numbers and sizes of lipid droplets. Brightfield images of 3T3-L1 cells were evaluated for A) number of droplets, and B) average droplet size via ImageJ after exposure to Profile 3 at the 1X, 50X, and 500X concentrations as well as a single exposure of BDE47 (500X).

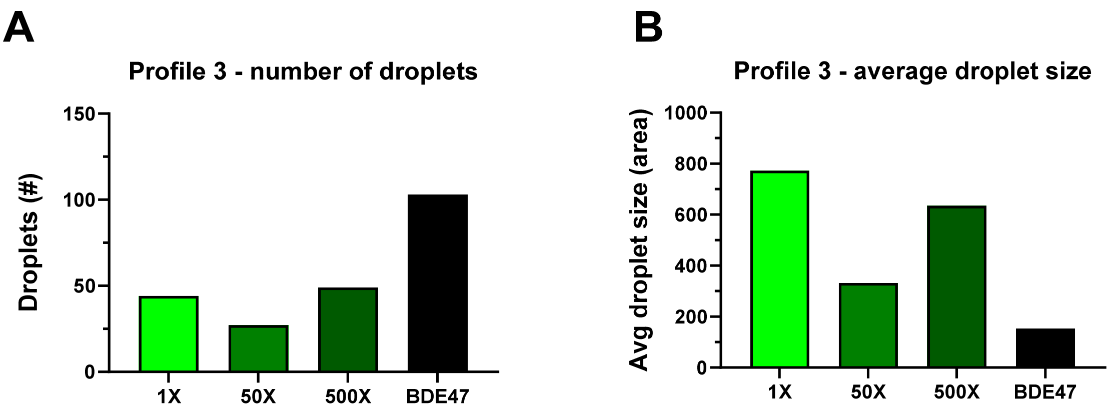

Figure S7. Profile 2 at the 500X concentration and PCB153 at the 500X concentration have the same number of lipid droplets, but different lipid droplet sizes. Brightfield images of 3T3-L1 cells were evaluated for A) number of droplets, and B) average droplet size via ImageJ after exposure to Profile 2 at the 1X, 50X, and 500X concentrations as well as a single exposure of PCB153 (500X).

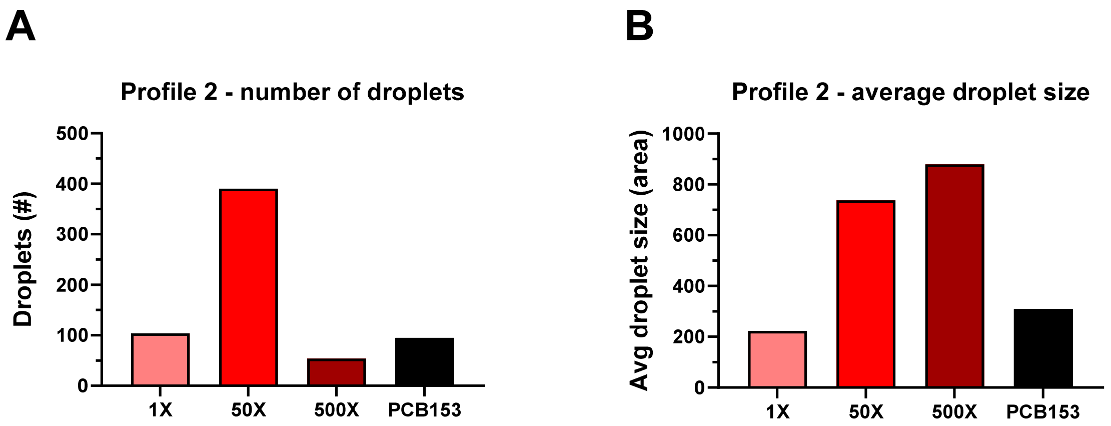

Figure S8. Exposure to Profile mixture 3 at 500X, and PCB153 and BDE47 individually at 15 µM activates PPARα in HepG2 cells. HepG2 cells were exposed to DMSO (control), GW590735 (0.1 µM), A) Profiles 1-5 (500X), or B) single chemicals *p,p'*-DDE, PCB153, BDE47, and PFOS (15 µM), or C) PCB153 (500X), BDE47 (500X), PCB (500X) and BDE47 (500X) or Profile 3 (500X) for 24 hours. GW590735 served as the positive control. Data represented as mean ± SEM. Statistical significance ($p < 0.05$) was determined by one-way ANOVA and Fisher's LSD post hoc test (n = 6).

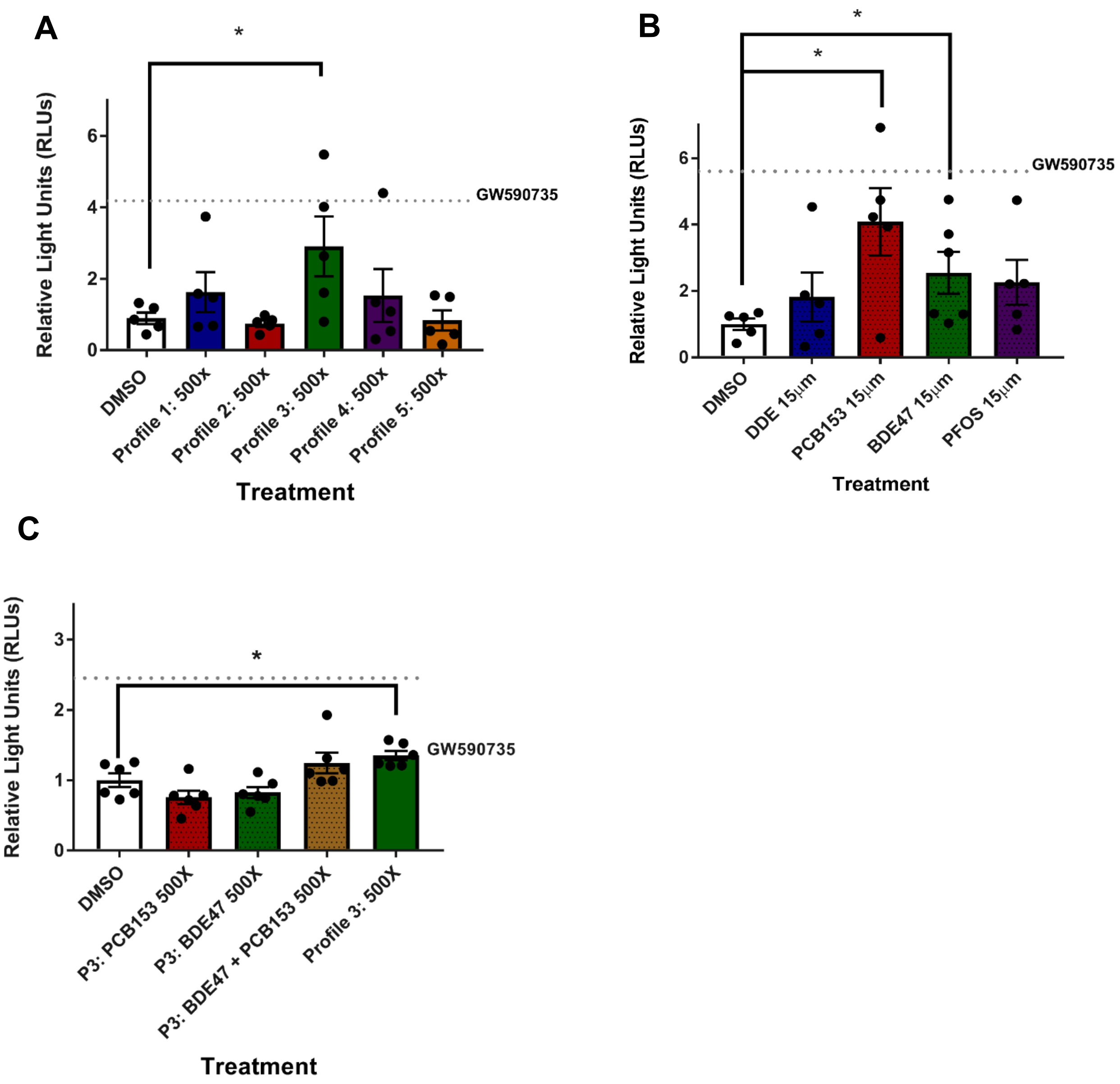

Supplement: Supplementary file 1 [file toxics-13-00975-s001.zip › toxics-3959323-supplementary.pdf]
